# The food-borne pathogen *Campylobacter jejuni* responds to the bile salt deoxycholate with countermeasures to reactive oxygen species

**DOI:** 10.1038/s41598-017-15379-5

**Published:** 2017-11-13

**Authors:** Nicholas M. Negretti, Christopher R. Gourley, Geremy Clair, Joshua N. Adkins, Michael E. Konkel

**Affiliations:** 1 School of Molecular Biosciences, College of Veterinary Medicine, Washington State University, Pullman, WA 99164-7520 USA; 2 Integrative Omics, Pacific Northwest National Laboratory, 902 Battelle Boulevard, Richland, Washington 99352 USA

## Abstract

Bile plays an important role in digestion, absorption of fats, and the excretion of waste products, while concurrently providing a critical barrier against colonization by harmful bacteria. Previous studies have demonstrated that gut pathogens react to bile by adapting their protein synthesis. The ability of pathogens to respond to bile is remarkably complex and still incompletely understood. Here we show that *Campylobacter jejuni*, a leading bacterial cause of human diarrheal illness worldwide, responds to deoxycholate, a component of bile, by altering global gene transcription in a manner consistent with a strategy to mitigate exposure to reactive oxygen stress. More specifically, continuous growth of *C*. *jejuni* in deoxycholate was found to: 1) induce the production of reactive oxygen species (ROS); 2) decrease succinate dehydrogenase activity (complex II of the electron transport chain); 3) increase catalase activity that is involved in H_2_O_2_ breakdown; and 4) result in DNA strand breaks. Congruently, the addition of 4-hydroxy-TEMPO (TEMPOL), a superoxide dismutase mimic that reacts with superoxide, rescued the growth of *C*. *jejuni* cultured in the presence of deoxycholate. We postulate that continuous exposure of a number of enteric pathogens to deoxycholate stimulates a conserved survival response to this stressor.

## Introduction

Bacterial pathogens that colonize the intestines of humans and animals must overcome the toxic components of bile. Bile contains a complex mixture of bile acids, including primary bile acids synthesized in the liver (cholic acid and chenodeoxycholic acid) and secondary bile acids produced from the resident microbiota (lithocholic acid and deoxycholic acid). Bile plays a critical role in the digestion and absorption of fats and in the excretion of waste products for the host^1^, while also posing as an important barrier to pathogenic bacteria. As such, pathogens that colonize the intestine respond to bile by adapting their protein composition. As one might predict, mutations in genes encoding lipopolysaccharide, the Tol proteins, efflux pumps, regulatory networks, and porins, have been found to affect bile resistance in enteric bacteria^2^. *Campylobacter jejuni*, a frequent bacterial cause of food-borne illness throughout the world, commonly colonizes the gut of chickens. Incompletely understood by investigators are the mechanisms used by pathogens, including *C*. *jejuni*, to respond to the stresses imposed by bile acids.

In susceptible individuals, *C*. *jejuni* causes fever, severe abdominal cramps, and diarrhea with blood and leukocytes in the stool^3^. *C*. *jejuni* is exposed to bile as it colonizes and proliferates in the gut. Studies have revealed that *C*. *jejuni* responds to bile by producing resistance proteins and synthesizing putative virulence factors/regulatory proteins. For example, the *Campylobacter* multidrug efflux pump (Cme) is known to play an important role in the resistance to bile salts in the chicken intestinal tract^4^. Raphael *et al*. (2005) reported that an orphan response regulator protein, termed CbrR, plays a role in bile resistance and chicken colonization^5^. Researchers have also found that the response to bile potentiates the virulence of *C*. *jejuni*. For example, bovine bile and deoxycholate, a component of bile, have been found to stimulate *flaA* promoter activity^6^. The importance of this finding is that FlaA is the major constituent of the flagellar filament^7,8^ and is important in the colonization of chicks^9,10^. In addition, Rivera-Amill *et al*. (2001) found that when cultured with deoxycholate, *C*. *jejuni* synthesizes proteins that are secreted from the bacterium through the flagellum and promote its invasion of epithelial cells^11^. Finally, Hu *et al*. (2013) identified *in vivo*-induced antigens using *C*. *jejuni* grown on deoxycholate plates^12^. Collectively, these studies provide strong evidence that *C*. *jejuni* adapt to deoxycholate.

The gut is a hostile environment for bacteria that are not adapted to the presence of bile. In fact, researchers have hypothesized that bile salts, including deoxycholate, might induce the production of reactive oxygen species (ROS) that act as a cumulative stressor to which bacteria respond^13,14^. While previous studies have focused on the changes in *C*. *jejuni* virulence gene expression in response to deoxycholate, less well-understood are the mechanisms that this bacterium uses to counteract the toxic effects of this bile acid. To address this knowledge gap, the resistance mechanisms of three *C*. *jejuni* clinical strains (81–176, F38011, and NCTC 11168) to a physiological concentration of the bile salt sodium deoxycholate was examined over time. A transcriptomic approach was used initially as a screen to identify if the clinical strains demonstrated a conserved response to deoxycholate. Experiments demonstrated that continuous exposure to deoxycholate elevates reactive oxygen species, downregulates genes encoding components of the electron transport chain, and increases the production of catalase in *C*. *jejuni*. Collectively, these studies demonstrate that continuous exposure to deoxycholate is acting as a “stressor”, ultimately resulting in DNA damage, and that *C*. *jejuni* adapts to this intrinsically occurring compound of the intestine by producing enzymes that mitigate ROS build-up.

## Results

### Similarities and differences in the genomic content amongst three *C*. *jejuni* clinical strains

The three *C*. *jejuni* clinical strains (81–176, F38011, and NCTC 11168, see Table 1) used in this study were recovered from individuals with gastroenteritis, and each tested positive for motility (Supplementary Fig. 1). Each strain was found to harbor unique genes (Supplementary Fig. 2). In addition, *C*. *jejuni* strain 81–176 carries two plasmids, pTet and pVir, and *C*. *jejuni* strain F38011 has a CJIE1-like insertion element. In summary, the genomic comparisons revealed a high degree of similarity amongst the three *C*. *jejuni* strains used in this study.

**Table 1:** Bacterial strains, plasmids, and oligonucleotides used in this study.

Strain	Isolation source
*C*. *jejuni* 81–176	Outbreak associated with raw milk^48^
*C*. *jejuni* F38011	Human case of diarrhea^49^
*C*. *jejuni* NCTC 11168	Human case of diarrhea, first sequenced strain^50^
**Oligo name**	**Sequence**
23 s rRNA probe #1	5′-AGGAATTTCGCTACCTTAGGACCGTTATAGTTA/3BioTEG/ -3′
23 s rRNA probe #2	5′-CTTTTCACCTTTCCCTCACGGTACT/3BioTEG/-3′
16 s rRNA probe #1	5′-CGTATTACCGCGGCTGCTGGCACG/3BioTEG/-3′
16 s rRNA probe #2	5′-AACATCTCACGACACGAGCTGACGAC/3BioTEG/-3′

### Deoxycholate alters *C*. *jejuni* growth in broth

**Figure 1:**
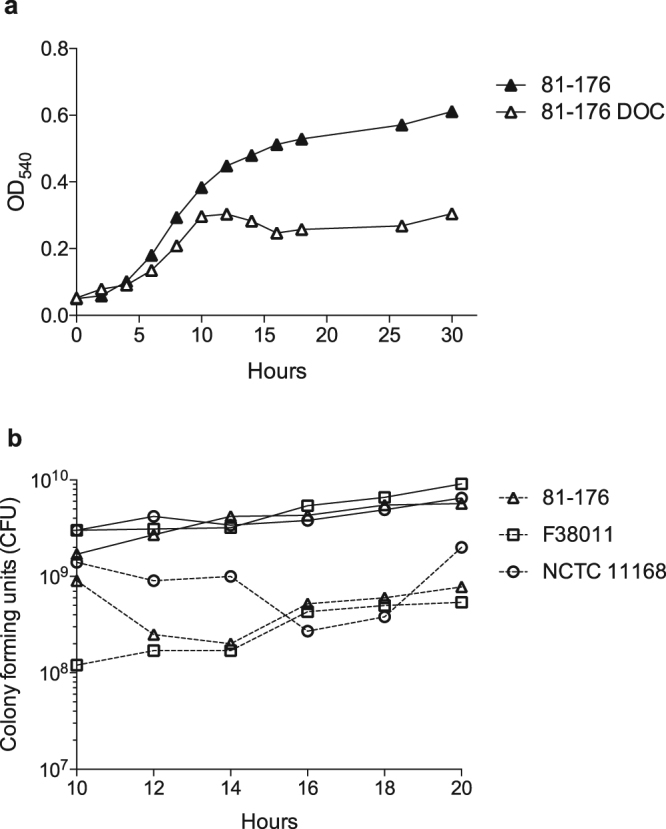
Incubation of *C*. *jejuni* in medium supplemented with deoxycholate impairs growth. Panels: (**a**) Shown is a representative assay plotting the Optical Density (OD_540_) of *C*. *jejuni* grown in MH broth (▴) and in MH broth supplemented with 0.05% deoxycholate (▵). (**b**) Shown is a representative assay plotting colony forming units (CFU) of all three *C*. *jejuni* strains 81–176 (▵), F38011 (▫), and NCTC 11168 (◦) grown in MH broth (solid lines) or MH broth with 0.05% deoxycholate (dashed lines). The experiments were repeated three times to ensure reproducibility.

We compared the growth of *C*. *jejuni* strains 81–176, F38011, and NCTC 11168 in MH broth and MH broth supplemented with a physiological concentration of deoxycholate (0.05% w/v). A noticeable decrease in the optical density (OD_540_) was observed for *C*. *jejuni* strain 81–176 after 12 hours of incubation in 0.05% deoxycholate compared with the growth of the bacteria in MH broth without deoxycholate (Fig. 1a). A similar observation was made for *C*. *jejuni* strains F38011 and NCTC 11168 grown in MH broth supplemented with deoxycholate versus MH broth alone (not shown). Viable bacteria (CFU/mL) were determined every two hours from 10 to 20 hours post-inoculation. Noteworthy is the decrease in CFUs for all three *C*. *jejuni* clinical strains grown in MH broth supplemented with deoxycholate versus that of the bacteria cultured in MH broth alone after 12 hours of growth (Fig. 1b). While the CFUs were observed to fluctuate (increase and decrease) for each culture between the 10 and 20 hours of incubation, the CFUs were very similar for all three *C*. *jejuni* strains at the 16 and 18 hour time points (Fig. 1b). The decrease in *C*. *jejuni* growth observed in the MH broth with deoxycholate was not due to changes in the pH of the growth medium, as the pH of the liquid cultures (MH and MH broth with deoxycholate) was within 0.2 units of a pH 7 over the course of the entire incubation period (not shown). Obvious from these experiments was that growth in medium with deoxycholate suppresses *C*. *jejuni* growth.

### Conserved *C*. *jejuni* gene responses to deoxycholate

**Figure 2:**
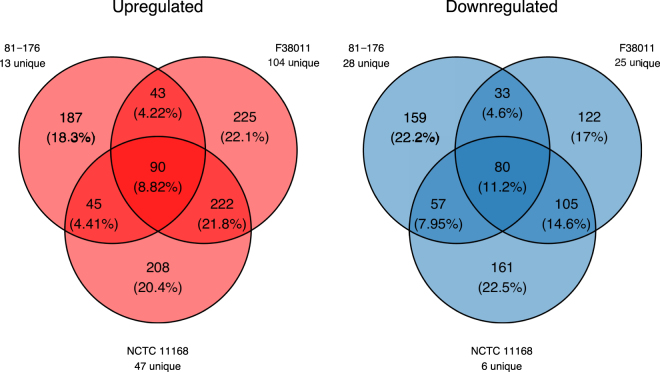
Deoxycholate alters gene expression in *C*. *jejuni*. Comparison of upregulated or downregulated genes from three *C*. *jejuni* strains (81–176, F38011, and NCTC 11168) grown for 16 or 18 hours in culture medium supplemented with deoxycholate. Genes that were significantly upregulated or downregulated had a Benjamini-Hochberg adjusted *p* value of less than 0.1 (*q* < 0.1) as determined by a Wald test implemented in DESeq 2. Genes that are unique to a given strain are indicated.

RNA-Seq experiments were performed using RNA extracted from the three *C*. *jejuni* strains to identify conserved changes (see Fig. 2 and Supplementary Figs. 3 and 4). Compared to the gene expression profile of each corresponding *C*. *jejuni* strain grown in MH broth for 12 hours, there were 90 genes upregulated and 80 genes downregulated in the presence of deoxycholate for all three strains at 16 and 18 hours (Fig. 2). These genes were grouped by Clusters of Orthologous Groups (COG) categories in order to identify trends in how *C*. *jejuni* responds to the stress of prolonged culture in deoxycholate (Table 2, Supplementary Table 1). Based on the observation that *C*. *jejuni* growth is impaired in deoxycholate, it is not surprising that deoxycholate causes downregulation of a large number of genes belonging to the category of energy production and conversion (see Table 2 and Supplementary Table 1). Specifically, central metabolic processes are downregulated. There are 16 downregulated genes that encode components of the electron transport chain [11 of 14 subunits of NADH dehydrogenase (complex I), 1 of 3 subunits of succinate dehydrogenase (complex II), and all four subunits of cytochrome C oxidase (complex IV)], and 8 downregulated components of the tricarboxylic acid cycle (fumarate reductase, succinyl-CoA synthetase, all three subunits of 2-Oxoglutarate: Acceptor oxidoreductase, aconitate hydratase, pyruvate-flavodoxin oxidoreductase, and citrate synthase). Moreover, the *katA* gene, which encodes the ROS detoxification enzyme catalase, was upregulated in response to deoxycholate. Taken together, we postulated that deoxycholate might induce an ‘oxidative stress response’. Given that *Campylobacter* bacteria, as members of the Epsilonproteobacteria, have fewer genes and regulatory pathways compared to Gammaproteobacteria, including *Escherichia coli* and *Salmonella enterica* subspecies Typhimurium, we decided to focus our efforts on the possibility that *C*. *jejuni* was responding to deoxycholate in a previously uncharacterized manner.

**Table 2:** Clusters of Orthologous Groups (COG) categories for differentially expressed genes. Indicated in bold are the two COGs that are significantly enriched. COG categories for upregulated genes.

Genes	COG category	Enrichment p-value (FDR*)
	COG categories for upregulated genes	
11	J: Translation, ribosomal structure and biogenesis	0.35 (0.87)
9	M: Cell wall/membrane/envelope biogenesis	0.32 (0.87)
9	E: Amino acid transport and metabolism	0.37 (0.87)
7	R: General function prediction only	0.15 (0.87)
6	L: Replication, recombination and repair	0.14 (0.87)
6	H: Coenzyme transport and metabolism	0.35 (0.87)
6	P: Inorganic ion transport and metabolism	0.23 (0.87)
5	F: Nucleotide transport and metabolism	0.18 (0.87)
5	I: Lipid transport and metabolism	0.09 (0.87)
4	O: Posttranslational modification, protein turnover, chaperones	0.64 (1)
4	G: Carbohydrate transport and metabolism	0.32 (0.87)
2	D: Cell cycle control, cell division, chromosome partitioning	0.34 (0.87)
6	Other **	N/A
10	Uncategorized	N/A
	COG categories for downregulated genes	
39	**C: Energy production and conversion**	<0.05 (<0.05)
11	**N: Cell motility**	<0.05 (<0.05)
10	E: Amino acid transport and metabolism	0.19 (1)
3	G: Carbohydrate transport and metabolism	0.52 (1)
2	J: Translation, ribosomal structure and biogenesis	1 (1)
2	M: Cell wall/membrane/envelope biogenesis	1 (1)
2	O: Posttranslational modification, protein turnover, chaperones	0.94 (1)
2	I: Lipid transport and metabolism	0.7 (1)
5	Other **	N/A
4	Uncategorized	N/A

*False Discovery Rate (FDR): Benjamini-Hochberg adjusted *p*-values.

**COG categories with one member were combined into ‘other’.

The incubation of each *C*. *jejuni* clinical strain in medium supplemented with deoxycholate reproducibly resulted in a cessation of growth compared to the growth of the bacteria cultured in MH broth alone (Fig. 1). Based on this observation, we questioned whether deoxycholate induced ‘stationary phase’ with a gene expression profile similar to *C*. *jejuni* grown in MH broth alone for a longer period of time. To address this question, RNA was extracted from *C*. *jejuni* strains 81–176, F38011, and NCTC 11168 grown in MH broth until stationary phase (24 hours) and RNA-Seq was performed. The gene expression profile from stationary phase *C*. *jejuni* was distinct from the gene expression profile of *C*. *jejuni* grown in deoxycholate for 16 and 18 hours (not shown). Based on these findings, we concluded that *C*. *jejuni* growth in deoxycholate results in a unique response, different from that of ‘stationary phase’.

### Deoxycholate triggers *C*. *jejuni* stress responses

**Figure 3:**
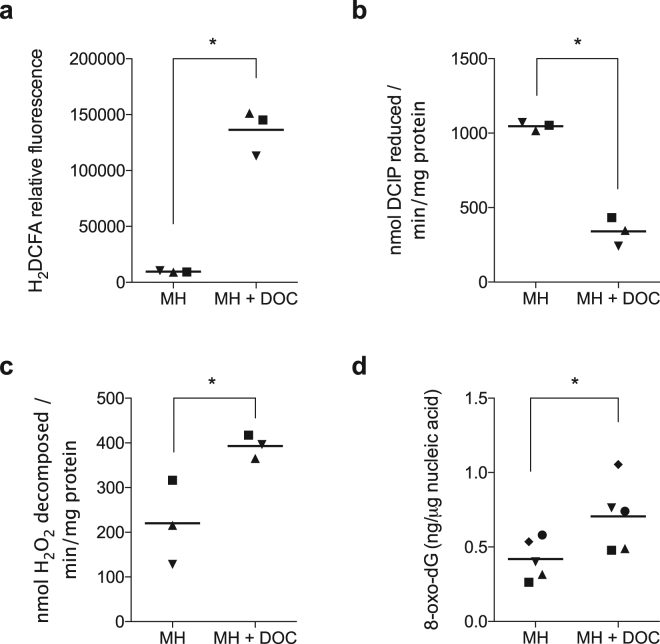
Growth of *C*. *jejuni* strain 81–176 in deoxycholate for 18 to 20 hours increases oxidative stress, decreases the activity of complex II of the electron transport chain, and induces 8-oxo-2′-deoxyguanosine (8-oxo-dG) lesions consistent with DNA damage from ROS. Panels: (**a**) The levels of intracellular reactive oxygen species were determined by incubating *C*. *jejuni* with 2′,7′-dichlorodihydrofluorescein diacetate (H_2_DCFA), a redox-sensitive dye that becomes trapped within a cell and fluoresces when oxidized. (**b**) The activity of succinate dehydrogenase (complex II) was measured by the rate of 2,6-dichlorophenolindophenol (DCIP) reduction in the presence and absence of succinate. (**c**) The rate of H_2_O_2_ decomposition of cellular lysates was determined using a dichromate reduction assay, as described in the ‘Materials and Methods’. (**d**) The amount of 8-oxo-dG in *C*. *jejuni* nucleic acid was determined by ELISA. For each assay, individual biological replicates are represented as different shapes. Significant differences between MH broth alone (MH) and MH broth with 0.05% deoxycholate (MH + DOC) were determined using a Student’s t-test and are indicated by the asterisk (*p < 0.05).

Based on the RNA-Seq data, continuous exposure to deoxycholate was hypothesized to increase the production of superoxide radicals, resulting in decreased electron transport chain (ETC) activity, a buildup of H_2_O_2_, and possibly, DNA damage. To test this hypothesis and to determine if *C*. *jejuni* counteracts these insults, the levels of intracellular ROS, the activity of complex II of the ETC, and the activity of catalase from *C*. *jejuni* grown with and without deoxycholate were measured. ROS was measured by the fluorescence of oxidized 2′,7′-dichlorodihydrofluorescein diacetate. A 12.2-fold increase in intracellular ROS was observed in *C*. *jejuni* cultured in MH broth supplemented with 0.05% deoxycholate when compared to bacteria grown in MH broth alone (Fig. 3a). The activity of complex II (succinate dehydrogenase) in cell lysates was measured by the rate of 2,6-dichlorophenolindophenol (DCIP) reduction in the presence and absence of succinate. The activity of complex II was determined to be decreased 2.9-fold in culture in MH broth with 0.05% deoxycholate compared to MH broth alone (Fig. 3b). Catalase activity was measured by determining the rate of H_2_O_2_ decomposition after the addition of a cellular lysate. We found that catalase (KatA) activity was increased 1.8-fold in MH broth supplemented with deoxycholate compared to MH broth alone (Fig. 3c). In summary, *C*. *jejuni* grown in the presence of deoxycholate have elevated levels of ROS, decreased ETC complex II activity, and increased catalase activity. These biochemical assays demonstrate that *C*. *jejuni* respond to deoxycholate stress by slowing metabolic activity and producing increased levels of ROS detoxification enzymes. In total, these strategies mitigate oxidative stress.

### Deoxycholate induces 8-oxo-dG lesions and double-strand breaks

**Figure 4:**
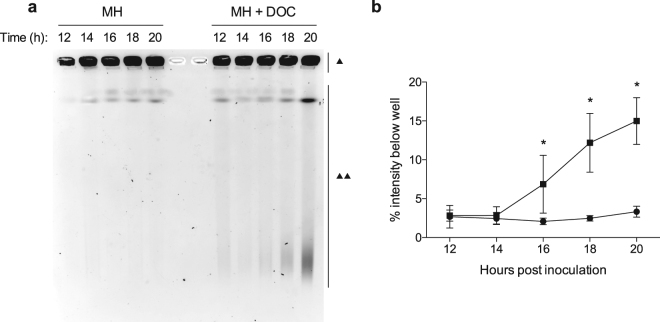
Growth in deoxycholate causes DNA double strand breaks (DSB). Panels: (**a**) Representative image showing a pulsed-field gel of genomic DNA from *C*. *jejuni* strain 81–176 cultured in MH broth (left side of gel) and from *C*. *jejuni* cultured in MH broth with 0.05% (w/v) deoxycholate (DOC, right side of gel). Bacterial samples were collected after the various periods of incubation indicated (hours) and processed as described in ‘Materials and Methods’. Chromosomal DNA was visualized by ethidium bromide staining after pulsed-field gel electrophoresis. The single triangle (▴) indicates intact DNA in the well, and two triangles (▴▴) indicate broken DNA. Contrast is adjusted to observe DNA fragments, see Supplementary Fig. 5 for the original image. (**b**) The intensity of each lane was quantified for samples collected from MH (▪) and from MH + DOC (⦁), and the total percentage of the DNA that left the well (broken DNA) was plotted. The mean ± the standard deviation for three biological replicates is shown. Significance between samples (MH and MH + DOC) at each time point was determined by one-way ANOVA followed by Sidak’s multiple comparisons test and are indicated by the asterisk (*p < 0.05).

ROS are known to result in DNA damage. 8-oxo-deoxyguanosine (8-oxo-dG) is one of the predominant forms of free radical-induced oxidative lesions and has been widely used by scientists as a biomarker for oxidative stress^15,16^. We postulated that the growth of *C*. *jejuni* in medium supplemented with deoxycholate would result in an increased amount of 8-oxo-dG lesions. To test this hypothesis, we measured the amount of 8-oxo-dG in DNA purified from *C*. *jejuni* grown with or without deoxycholate using an ELISA. This experiment revealed that *C*. *jejuni* grown in medium supplemented with deoxycholate results in a 1.7-fold increase of 8-oxo-dG when compared to the DNA extracted from *C*. *jejuni* grown in MH medium alone (Fig. 3d). Concordantly, double strand breaks (DSB) arise when the replication or transcription machinery encounter ROS-induced lesions. As such, we assessed whether *C*. *jejuni* growth in medium supplemented with deoxycholate results in DNA double strand breaks by pulsed-field gel electrophoresis (PFGE). *C*. *jejuni* growth in deoxycholate-supplemented medium resulted in fragmentation of *C*. *jejuni* DNA, as judged by the increase in the intensity of the smear at the bottom of the gel (Fig. 4). Based on these findings, we concluded that *C*. *jejuni* growth in deoxycholate-supplemented medium results in DNA double strand breaks.

### The addition of an antioxidant compound promotes *C*. *jejuni* growth in deoxycholate-supplemented medium

**Figure 5:**
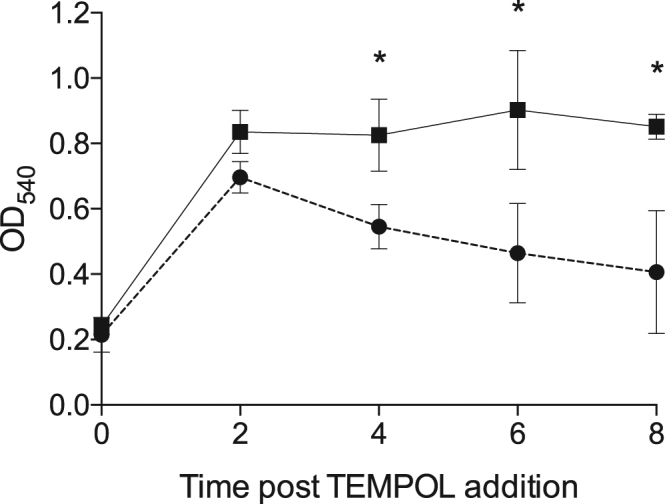
Addition of ROS scavenger 4-hydroxy-TEMPO (TEMPOL) enhances *C*. *jejuni* growth in deoxycholate (DOC). *C*. *jejuni* strain 81–176 was grown for 20 hours in MH broth with 0.05% (w/v) DOC and then the culture was divided into new flasks containing fresh MH broth with either 0.1% DOC (⦁) or 0.1% DOC supplemented with 0.1 mM TEMPOL (▪). Optical density was determined immediately after splitting the culture (time zero) and every two hours for eight hours. Three biological replicates ± standard deviation are plotted for each time point. Significance between samples (DOC and DOC + TEMPOL) at the 4, 6, and 8 hour time points was determined by one-way ANOVA followed by Sidak’s multiple comparisons test and are indicated by the asterisk (*p < 0.05).

Two possible reasons for the reduced growth of *C*. *jejuni* in deoxycholate-supplemented medium versus growth in MH medium alone are decreased activity of the electron transport chain or oxidative damage of the DNA. We favored the hypothesis that the change in *C*. *jejuni* growth was due to the oxidative damage of DNA. If this is indeed the case, we postulated that it should be possible to protect *C*. *jejuni* from the deleterious effects of ROS in the cell by adding an antioxidant compound to the growth medium^17^. 4-hydroxy-TEMPO (TEMPOL) was chosen for these assays because it is a superoxide dismutase mimic that reacts with superoxide (O_2_^−^)^18^. Addition of 0.1 mM of TEMPOL to the deoxycholate-supplemented culture after 20 hours of incubation increased the rate of growth and terminal optical density of *C*. *jejuni* grown in deoxycholate (Fig. 5). Based on this finding, we concluded that enhanced deoxycholate protection can be exerted via the reduction of oxidative damage.

## Discussion

Life in the presence of oxygen inevitably results in exposure to reactive oxygen species, including singlet oxygen, superoxides, peroxides, and hydroxyl radicals. Generation of ROS can result from a wide range of cellular and environmental processes from cellular respiration to photo-oxidation^19^. A major source of ROS in bacteria are the redox enzymes mediating oxidative phosphorylation through the electron carriers^20^. Known is that bile interacts with cellular membranes, resulting in altered membrane permeability. This altered membrane permeability has been shown to impede the proper function of the electron transport chain in mitochondria and may contribute to production of reactive oxygen species in mammalian cells^21^.

**Figure 6:**
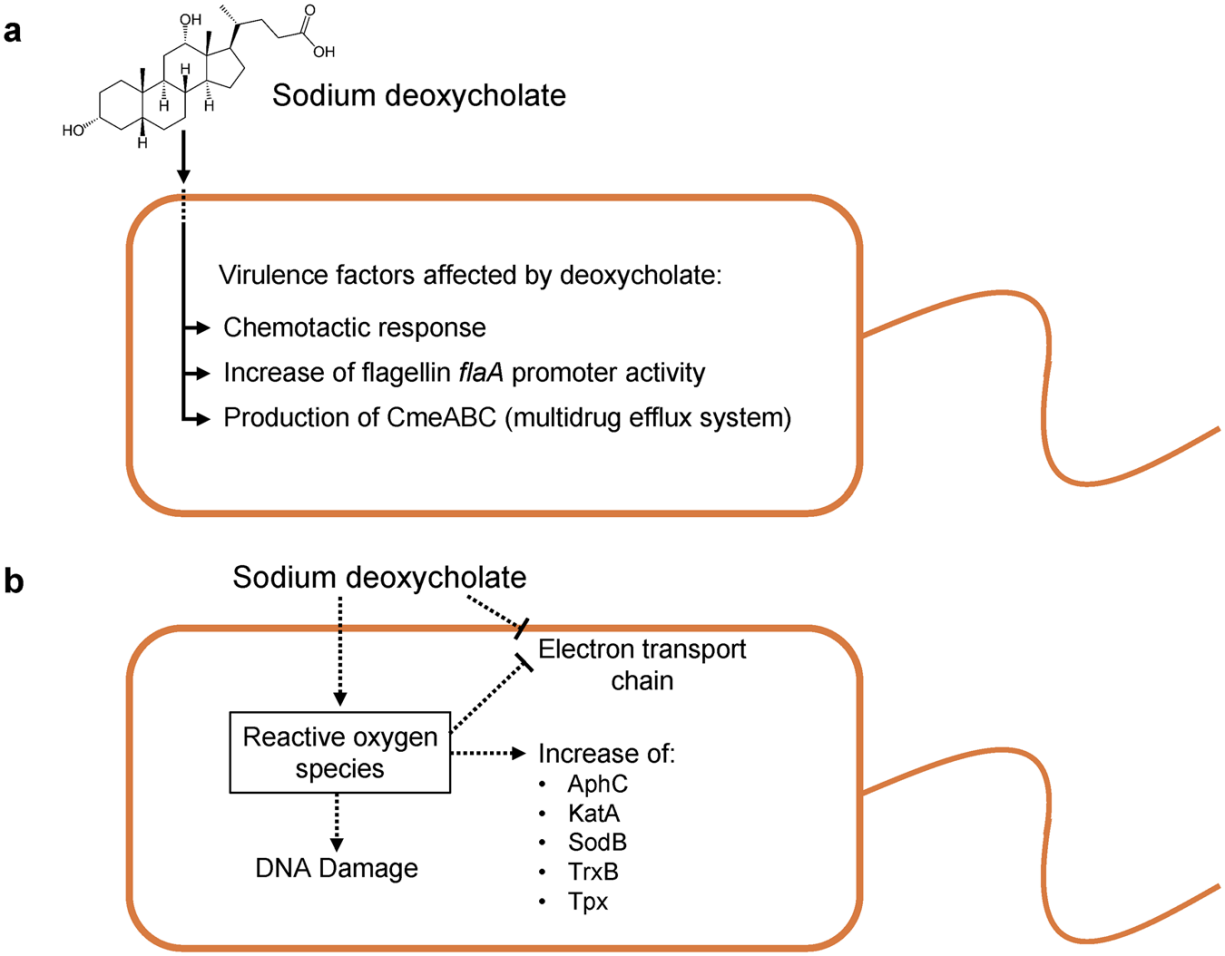
*C*. *jejuni* strains display a conserved response to the cumulative toxic effects of growth in deoxycholate. *C*. *jejuni* are exposed to deoxycholate when they enter the gut. Panel **a** shows the initial response of *C*. *jejuni* to deoxycholate, as demonstrated by previous studies. Panel **b** highlights the response of *C*. *jejuni* to the cumulative toxic effects of growth in deoxycholate. The model in Panel **b** is based on RNA-Seq and proteomics is supported by enzymatic assays that assessed intracellular levels of reactive oxygen species, the activity of complex II of the electron transport chain, and catalase activity. We propose that the conserved responses observed with the three *C*. *jejuni* strains used in this study will be broadly applicable to other *Campylobacter* strains and to other gut pathogens.

We sought to identify *C*. *jejuni* genes that are differentially expressed in response to a physiological concentration of deoxycholate. We chose to use RNA-Seq for this study owing to its ability to detect low-abundance RNAs and high dynamic range. In total, we found that continuous growth of *C*. *jejuni* in deoxycholate: 1) induces the production of reactive oxygen species; 2) decreases succinate dehydrogenase activity (complex II of the ETC); 3) increases catalase activity; and 4) causes DNA strand breaks (discussed below). Figure 6 shows a model whereby deoxycholate, acting as a stressor, causes *C*. *jejuni* to adapt in order to survive.

In this study, a significant increase in intracellular ROS was observed when *C*. *jejuni* was cultured in medium supplemented with deoxycholate versus without deoxycholate. Noteworthy is that *C*. *jejuni* is a microaerophilic bacterium, and thus by definition, it requires oxygen to grow but is sensitive to atmospheric oxygen. Previously recognized is that *C*. *jejuni* uses multiple strategies, which can be synergistic, to mitigate the deleterious effects of ROS. For example, *C*. *jejuni* can utilize AhpC (alkyl hydroperoxide reductase), Bcp (thiol peroxidase), Dps (bacterioferritin), KatA (catalase), MsrA/B, SodB (superoxide dismutase), Tpx (thiol peroxidase) and Cj1386 (an ankyrin-containing protein) to reduce the concentration of ROS^22–29^. The RNA-Seq experiments performed in this study revealed that *C*. *jejuni* upregulated the expression of *katA*, and proteomic experiments, done in parallel, revealed increased levels of AhpC, SodB, and Tpx in *C*. *jejuni* in response to deoxycholate (not shown). The transcriptomic data also revealed that the expression of genes encoding complex I and complex II components of the ETC were downregulated, which could translate into fewer ROS being produced in the bacterial cell. The reason that culturing *C*. *jejuni* in medium with deoxycholate triggers the production of three major detoxification enzymes (KatA, AhpC, and SodB) and the downregulation of genes encoding complex I and complex II ETC components may be related to a strategy to minimize DNA damage. The oxidation of guanine to 8-oxo-deoxyguanosine in *C*. *jejuni* in response to deoxycholate is especially noteworthy because it is one of the most abundant lesions generated in mammalian cells from the interaction of reactive oxygen species with DNA and because this base lesion, if not repaired, can result in mutations. Further evidence supporting the hypothesis that deoxycholate acts as a stressor was based on the finding that TEMPOL, a superoxide dismutase mimic, rescued the growth of *C*. *jejuni* in medium supplemented with deoxycholate.

Regardless of the particular study, it is evident that bile and the components of bile induce biological damage that *C*. *jejuni* must adapt to in order to survive. Kreuder *et al*.^30^ recently evaluated the transcriptional response of a clonal isolate from a *C*. *jejuni* strain, responsible for sheep abortion, to ovine bile. Although significant differences are apparent in the experimental designs of their study and ours, the gene expression profiles share some similarities. More specifically, nearly half of the genes observed to be downregulated in both studies belong to the COG category C (energy production and conversion). Palyada *et al*.^31^ examined the response of *C*. *jejuni* to oxidative stressors, including H_2_O_2_, by microarray analysis. Noteworthy is that *katA and uvrC* were both observed to be upregulated in the Palyada study with H_2_O_2_^31^, in the Kreuder study with ovine bile^30^, and by all three *C*. *jejuni* clinical strains used in our study with deoxycholate. The *uvrC* gene encodes a protein involved in DNA repair. Taken together, *C*. *jejuni* share similarities in gene expression in response to oxidative and bile stresses.

Previous work has revealed that, at a minimum, the *C*. *jejuni* ferric uptake regulator (Fur), the peroxide stress regulator (PerR), and the *Campylobacter* oxidative stress regulator (CosR), are involved in the response of *C*. *jejuni* to oxidative stress. While the results of this study indicate that *C*. *jejuni* can sense (directly or indirectly) and respond to deoxycholate (which increases the production of oxidative stress), they do not, *per se*, provide new insight into the molecular mechanism(s) by which *C*. *jejuni* are altering gene expression/protein production. Nevertheless, several response regulators have been implicated in altering gene expression in response to oxidative stress (e.g., PerR and Fur^32^) or components of bile (e.g., CbrR^5^ and CmeR^33^), and it is plausible these regulators contribute to the downstream changes in gene expression observed in this study. For example, it is well documented that Fur, PerR, and CosR are all involved in *katA* regulation^34^. Moreover, isolates that have mutations in the genes encoding the KatA, AhpC, and SodB enzymes are attenuated in chicken colonization^31^. Taken together, these findings demonstrate that *C*. *jejuni* must combat oxidative stress in the natural host.

Each of the *C*. *jejuni* strains used in this study contained unique genetic elements. *C*. *jejuni* strain 81–176 contains the pTet plasmid and pVir plasmid, and *C*. *jejuni* strain F38011 contains the CJIE1 prophage. No differences were noted in the expression profiles and responses of the genes on the pTet and pVir plasmids in response to deoxycholate. However, 50 genes were upregulated and four genes were downregulated within the *C*. *jejuni* strain F38011 CJIE1 prophage element (a Mu-like phage) in response to deoxycholate. Despite this observation, phage particles were not observed in samples prepared from MH broth cultures supplemented with deoxycholate by transmission electron microscopy (not shown). We previously noted an increase in CJIE1 gene expression in response to deoxycholate by microarray analysis^35^. Moreover, Clark and coworkers^36,37^ detected prophage proteins in deoxycholate-supplemented cultures by 2D-LC-MS/MS analysis and observed that the presence of the prophage CJIE1 is associated with increased host cell adherence and invasion of *C*. *jejuni*. These preliminary findings warrant additional studies to determine the regulation of CJIE1 encoded genes and their role in bacterial fitness in stress conditions and within hosts.

The chromosomes of bacterial cells are continually acquiring DNA lesions of oxidative origin that must be repaired for replication. This study is the first to compare the response of three *C*. *jejuni* clinical strains to a physiological concentration of the bile salt deoxycholate over time. When *C*. *jejuni* enters the intestine, it immediately encounters bile. Informed by changes in gene expression, biochemical assays were performed that confirmed that deoxycholate exposure results in an oxidative assault that *C*. *jejuni* must mitigate in order to survive. The data show a clear relationship between deoxycholate-mediated oxidative stress and significant damage to *C*. *jejuni* DNA. Beyond *C*. *jejuni*, all enteric pathogens are exposed to this oxidative assault in the intestine. As such, other enteric pathogens likely utilize similar adaptive mechanisms to defend against the toxic components of bile and to minimize DNA damage.

## Materials and Methods

### Bacterial strains, plasmids, and oligonucleotides

Table 1 lists the strains, plasmids, and oligonucleotides used for these studies. *C*. *jejuni* were routinely cultured on Mueller-Hinton (MH) agar (Difco Brand, BD Biosciences, Sparks, MD) containing 5% citrated bovine blood (MHB agar) under microaerobic (5% O_2_, 10% CO_2_, 85% N_2_) conditions at 37 °C.

### Genome analysis

Genome sequences from *C*. *jejuni* strains 81–176 [chromosome sequence: (NC_008787), plasmid sequences: pTet (NC_008790) and pVir (NC_008770)], F38011 (CP006851), and NCTC 11168 (AL111168) were obtained from Genbank. Mauve (snapshot_2015-02-25) was used for whole genome nucleotide alignments and identification of homologous genes^38^.

### Bacterial growth curves and sample collection for RNA-Seq

*C*. *jejuni* strains were inoculated at an OD_540_ of 0.05 in 250 mL of MH broth and MH broth supplemented with 0.05% (w/v) sodium deoxycholate in 500 mL flasks and incubated at 37 °C under microaerobic conditions with orbital shaking (220 rpm). To ensure that the *C*. *jejuni* strains were treated identically to each other, each experiment consisted of culturing all three *C*. *jejuni* strains in MH and MH deoxycholate side-by-side in the same chamber. Multiple analyses were performed for each culture at the time points indicated in the text. Optical density was determined by measuring the absorbance at a wavelength of 540 nm, CFU was determined by streaking serially-diluted samples on MH blood agar plates, and bacteria were pelleted and snap frozen in liquid nitrogen for RNA extraction.

### RNA extraction, rRNA depletion, and transcriptomic analysis

Total bacterial RNA was isolated using the Ambion Ribopure Bacteria kit and supplied DNAse (Thermo Fisher, Waltham, MA). Samples were collected from *C*. *jejuni* cultured in MH broth and MH deoxycholate at 12, 14, 16, 18, and 20 hours post-inoculation. In addition, samples were collected from *C*. *jejuni* cultured in MH broth at 24 hours post-inoculation. Depletion of rRNA was performed as described previously^39^. Briefly, 3′ biotinylated oligonucleotides with a tetraethylene glycol (TEG) spacer (Integrated DNA Technologies, Coralville, IA) were designed to hybridize with *C*. *jejuni* 16 S and 23 S rRNAs (Table 1). Two µg of each RNA sample was suspended in TES buffer (10 mM Tris, 1 mM EDTA, 1 M NaCl, pH 8.0) and mixed with 50 pmol of oligos. The samples were then incubated at 70 °C for 15 min followed by 37 °C for 15 min. TES equilibrated streptavidin coated agarose beads (GoldBio, Olivette, MO) were used to capture depletion oligos and bound rRNA. rRNA depletion was assessed with an Advanced Analytical Fragment Analyzer (Ankeny, IA). The cDNA libraries were generated with the Ion Total RNA-Seq Kit v2, sequencing beads were prepared using the Ion Chef™ System, and sequencing was performed on an Ion Proton™ Sequencer with a PI Chip (Thermo Fisher). Reads were mapped to the appropriate genome using Bowtie2 (version 2.2.5)^40^ and features counted with featureCounts (version 1.5.0)^41^. Differential expression analysis was performed using DESeq 2 (version 1.10.1)^42^. The 12 hour RNA-Seq data for each *C*. *jejuni* strain grown in MH broth alone was used as the baseline of gene expression, as this is the last time point at which the two *C*. *jejuni* cultures (MH and MH-deoxycholate) demonstrated similar optical densities. A Benjamini-Hochberg adjusted *p* value of less than 0.1 (*q* < 0.1) was chosen as a statistical cutoff. Two biological replicates were sequenced and analyzed for each experimental condition. Batch effects, as a result of different sequencing runs, were identified and corrected using RUVSeq. Genes were categorized using Cluster of Orthologous Groups (COG) categories automated by a custom R script, and enrichment was analyzed using Fisher’s exact test. Data analysis revealed that the RNA-Seq results were highly reproducible for a given strain and individual time point, as determined by unsupervised hierarchical cluster analysis by Poisson distance and principal component analysis.

### Evaluation of intracellular ROS levels

*C*. *jejuni* 81–176 was cultured in MH broth at 37 °C under microaerobic conditions with or without 0.05% sodium deoxycholate for 18 hours on an orbital shaker at 220 rpm. A 1 mL aliquot was suspended to an OD_540_ of 0.1 in MH broth and incubated with 20 µM of 2′,7′-dichlorodihydrofluorescein diacetate for 30 minutes, followed by centrifugation at 8,000 × *g* for 2 minutes, and resuspension in PBS. Fluorescence of a 200 µL aliquot was measured in a 96-well plate using a Victor X5 plate reader (PerkinElmer, Waltham, MA) with a 485 nm excitation filter and a 535 nm emission filter. The optical density of the sample in the plate was also determined using a 595 nm filter.

### Measurement of succinate dehydrogenase and catalase activity

Whole bacteria grown in MH broth or MH broth with 0.05% deoxycholate for 18 hours were recovered by centrifugation at 10,000 × *g* for 10 minutes and cell lysates were prepared by sonication on ice using 30 second pulses with 30 second pauses between pulses. Bicinchoninic acid (BCA) analysis was performed to determine protein concentration of *C*. *jejuni* whole cell lysates. The activity of electron transport chain complex II in *C*. *jejuni* whole cell lysates was determined as described by Brenner-Lavie *et al*.^43^. Catalase activity from *C*. *jejuni* whole cell lysates was determined using a solution of 800 µmoles H_2_O_2_ in PBS at room temperature as described by Sinha^44^. The amount of H_2_O_2_ in the reaction mixture was determined every minute for 10 minutes by mixing an aliquot with dichromate/acetic acid (5% K_2_Cr_2_O_7_/acetic acid 1:3). The optical density of the samples was measured at 562 nm, using a Bio-Tek ELx808IU microplate reader.

### PFGE

Pulsed-field gel electrophoresis was performed as described previously with some modification^45,46^. Briefly, *C*. *jejuni* F38011 was grown in MH or 0.05% deoxycholate for 12, 14, 16, 18, and 20 hr. Approximately 1 × 10^8^ CFU were suspended in PETT IV buffer (1 M NaCl, 10 mM Tris, 10 mM EDTA, pH 8.0) and embedded in agar plugs. Agar plugs were incubated for 2 hours at 37 °C in a buffer containing lysozyme and RNAse (0.1 mg/mL lysozyme, 20 µg/mL RNAse A, 1% N-lauroyl sarcosine, 100 mM EDTA, pH 8.0) followed by incubation overnight at 50 °C in ESP buffer (50 mM Tris-HCl, 50 mM EDTA, 1% N-lauroyl sarcosine, 0.5 mg/mL Proteinase K, pH 8.0). Agar plugs were washed twice in TE (10 mM Tris, 1 mM EDTA, pH 8.0) prior to loading in a 1% agarose gel.

### Recovery of growth by addition of 4-hydroxy-TEMPO (TEMPOL)

*C*. *jejuni* was cultured for 20 hours in MH broth with 0.05% deoxycholate, pelleted, and then suspended in fresh MH broth with 0.1% deoxycholate. The culture was split in two, 0.1 mM of TEMPOL (Sigma-Aldrich, St. Louis, MO) was added to one of the cultures and water (vehicle) was added to the other. The optical density was determined at the beginning of the assay, and every two hours for eight hours.

### Accession numbers

RNA-Seq data have been deposited in the NCBI Gene Expression Omnibus database with the identifier GSE89641. The mass spectrometry proteomics data have been deposited in the ProteomeXchange Consortium via the PRIDE^47^ partner repository with the dataset identifier PXD005306 and 10.6019/PXD005306.

## Electronic supplementary material

Supplementary Figures

Supplementary Table 1
