# Supplementary material for: The food-borne pathogen Campylobacter jejuni responds to the bile salt deoxycholate with countermeasures to reactive oxygen species
Source: Sci Rep. 2017 Nov 13;7:15455. doi: 10.1038/s41598-017-15379-5 (PMC5684402; doi:10.1038/s41598-017-15379-5)
Supplement: Supplementary file 1 — Supplementary Figures [file 41598_2017_15379_MOESM1_ESM.pdf]

The food-borne pathogen *Campylobacter jejuni* responds to the bile salt deoxycholate with countermeasures to reactive oxygen species

Nicholas M. Negretti[1], Christopher R. Gourley[1], Geremy Clair[2], Joshua N. Adkins[2], and Michael E. Konkel[1]*

[1]School of Molecular Biosciences, College of Veterinary Medicine, Washington State University, Pullman, WA, USA, 99164-7520.
[2]Integrative Omics, Pacific Northwest National Laboratory, 902 Battelle Boulevard, Richland, WA, USA, 99352.

*To whom correspondence should be addressed: Dr. Michael E. Konkel, Biotechnology Life Sciences Building, Room 447, Washington State University, Pullman WA 99164, Telephone: 509-335-5039; Email: konkel@vetmed.wsu.edu

Supplementary Methods

Proteomic sample preparation and LC-MS/MS analysis. For the proteomics analysis, cell pellets were lysed in 100 mM ammonium bicarbonate by bead beating, 5 times for 1 minute using 0.1 mm zirconia/silica beads. Urea and DTT were added to the samples to the concentration of 8 M and 5 mM, respectively. The samples were placed for 1 hour at 37°C. Then samples were diluted to obtain a final concentration of 1 M urea prior to trypsin digestion over 3 hours at 37°C. The resulting peptides were desalted using C18 SPE cartridges (Discovery C18, 1 mL, 50 mg, Sulpelco). The peptide concentrations were measured by BCA assay (Thermo Scientific). For each sample, 5 µl of 0.1 µg/µl was analyzed by reverse phase LC-MS/MS using a 100 minute gradient and Top-10 strategy on a QExactive Orbitrap mass spectrometer (Thermo Scientific). Protein digests were separated on a 4-column, custom-built, capillary LC system, as reported previously [1]. Mobile phases consisted of 0.1% formic acid in water (A) and 0.1% formic acid in 100% acetonitrile (B) with a 100 minute gradient. MS spectra (AGC 3×10^6) were collected in positive ionization mode from 400 to 2000 m/z at a resolution of 100 k as previously described [2].

Proteomic data analysis. Raw mass spectrometry data were searched with MS-GF+ against the RefSeq databases for *Campylobacter jejuni* subsp. *jejuni* strain F38011 (1,679 sequences), bovine trypsin, and human keratin sequences. The searching parameters were the following: semi-tryptic digestion, 20 ppm peptide mass tolerance, and methionine oxidation as variable modification. The identification of the MS/MS spectra was performed using MS-GF+. Spectral, peptide, and protein false discovery rate were set below 1%. Only proteins identified with at least two peptides were retained for identification. A spectral counting strategy was used for the protein quantification. Only the proteins with a minimum of 10 identified spectra in ≥75% percent of the samples from at least one condition were conserved for quantification. The data was log transformed and sum-normalized and imputed with the minimum protein expression value divided by 2. The t-tests, k-means clustering, PCA, and figures were generated using R 3.2.2.

References

1 Piehowski, P. D. *et al.* Sources of technical variability in quantitative LC-MS proteomics: human brain tissue sample analysis. *J Proteome Res* **12**, 2128-2137, doi:10.1021/pr301146m (2013).

2 Zhang, Q. *et al.* High and low doses of ionizing radiation induce different secretome profiles in a human skin model. *PLoS One* **9**, e92332, doi:10.1371/journal.pone.0092332 (2014).

Supplementary Figures

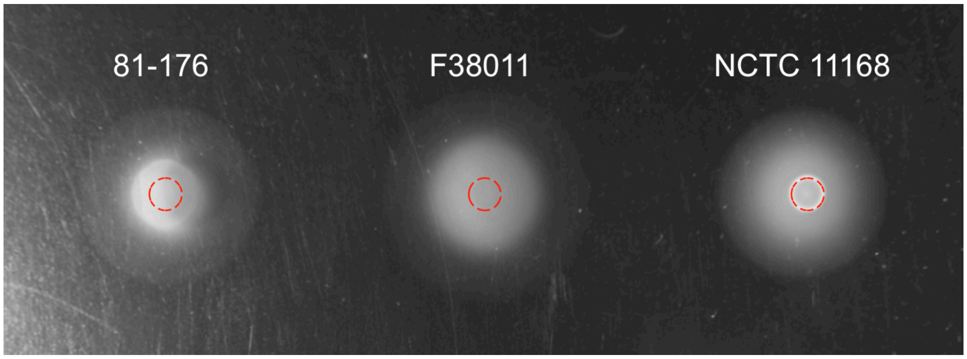

Supplementary Figure 1. The *C. jejuni* strains used in this study are motile. Normalized (OD_{540} of 1.0) suspensions of *C. jejuni* strains 81-176, F38011, and NCTC 11168 were spotted on soft agar plates and incubated overnight. The red circle indicates the location of the initial inoculation.

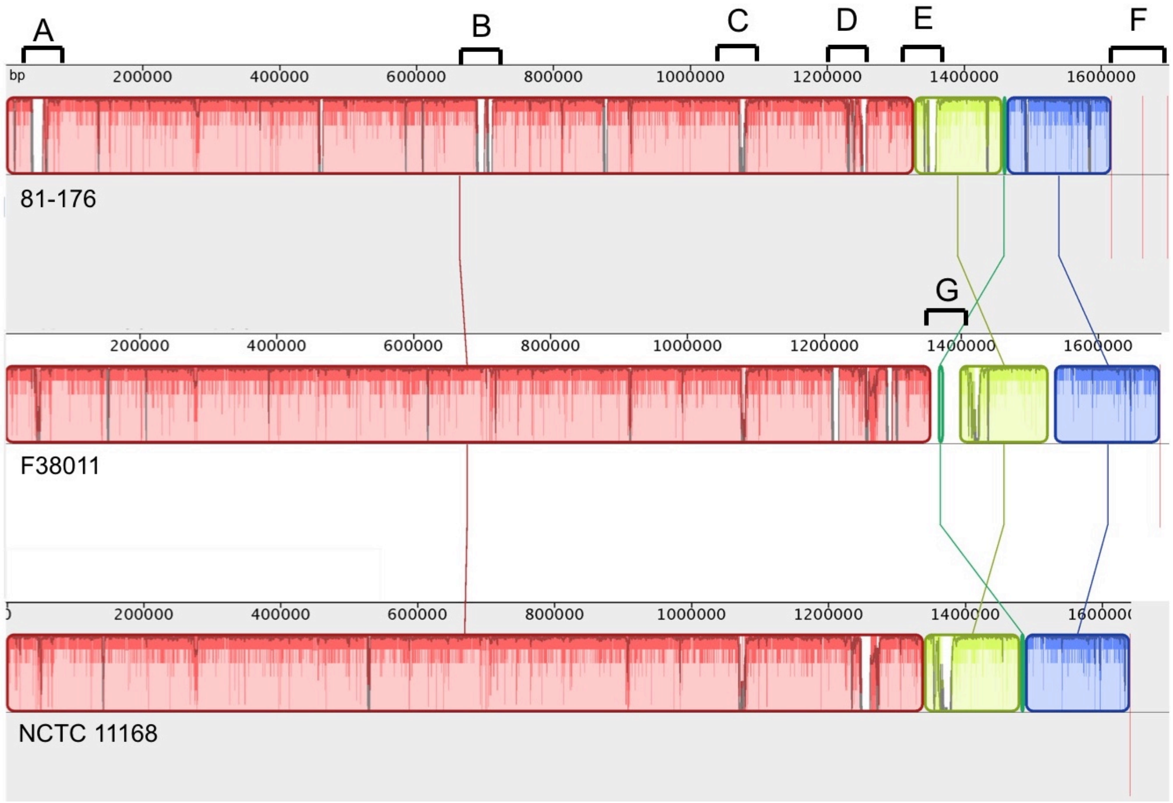

Supplementary Figure 2. Genome alignments show high similarity between the *C. jejuni* strains used in this study. The genome of *C. jejuni* strains 81-176, F38011, and NCTC 11168 were aligned and compared with progressiveMauve, with the default options except, "using seed families" was enabled and "seed weight" was set to 21. Each colored block represents a large region of homology between the strains, and the colors within the blocks represent the relative level of identity at that location (with white indicating lower identity). Regions of significant difference include: (A) membrane proteins and cytochrome C, (B) restriction modification and hypothetical proteins, (C) glycosyltransferase proteins, (D) flagellar related proteins, (E) glycosyltransferase and capsule related proteins, (F) pVir and pTet (present in *C. jejuni* strain 81-176), and (G) a CJIE1-like integration element (present in *C. jejuni* strain F38011). Each strain belongs to a different multilocus sequence type (MLST): *C. jejuni* 81-176 belongs to ST-604, *C. jejuni* F38011 belongs to ST-3644, and *C. jejuni* NCTC 11168 belongs to ST-43 (pubMLST database). In total, there were 1,420 genes common amongst all three strains and 142 shared between two strains. There were 218 genes unique to *C. jejuni* strain 81-176, 160 genes unique to *C. jejuni* strain F38011, and 103 genes unique to *C. jejuni* strain NCTC 11168. *C. jejuni* strain F38011 contained 60 genes that comprise the CJIE1-like prophage. While there is a very high degree of nucleotide identity among these three *C. jejuni*, only 68.9% of all of the predicted coding sequences are shared among all three strains.

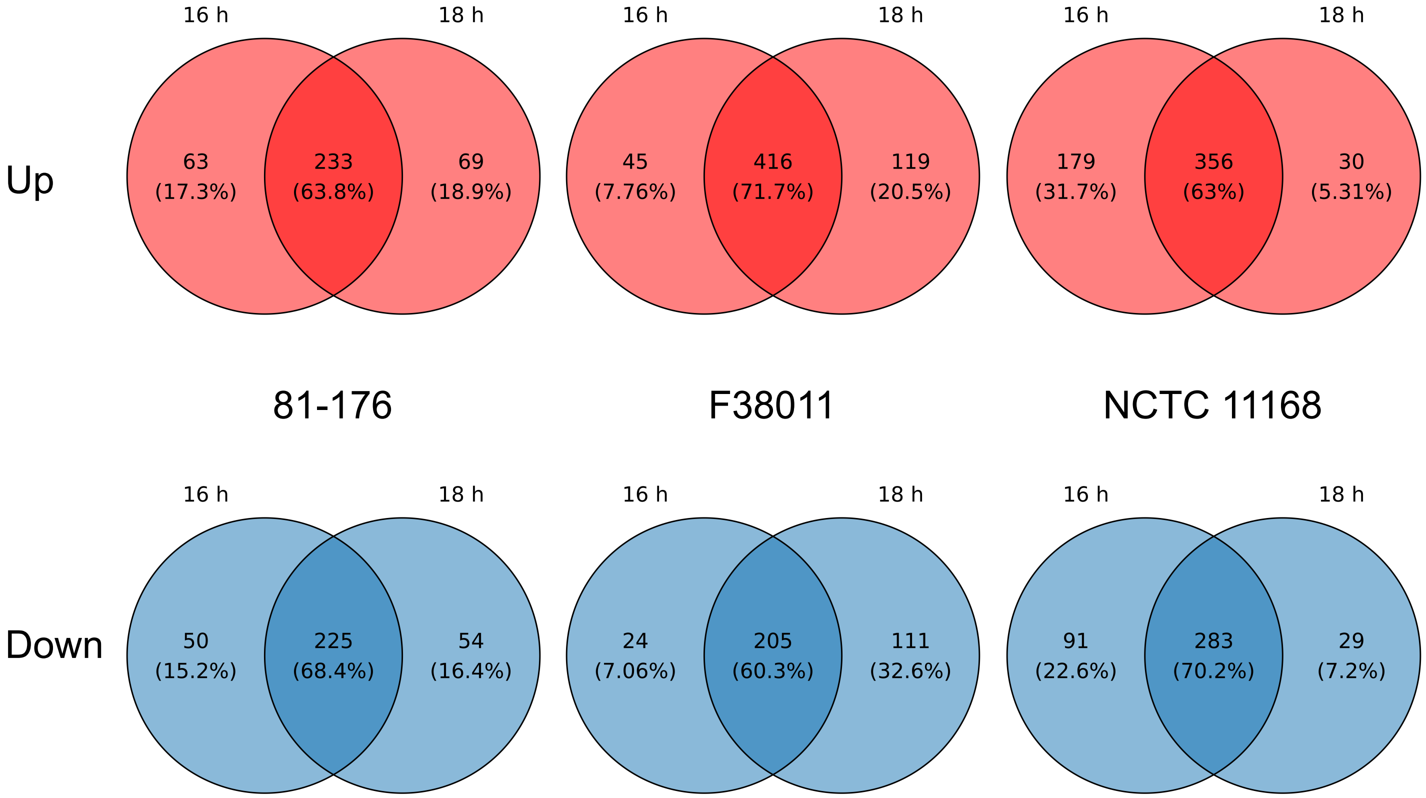

Supplementary Figure 3. Comparison of the number of genes upregulated and downregulated within a *C. jejuni* strain (81-176, F38011, and NCTC 11168) grown in MH with deoxycholate at 16 and 18 hours versus *C. jejuni* grown in MH for 12 hours. Genes that were significantly upregulated or downregulated had a Benjamini-Hochberg adjusted p value of less than 0.1 ($q < 0.1$) as determined by a Wald test implemented in DESeq2.

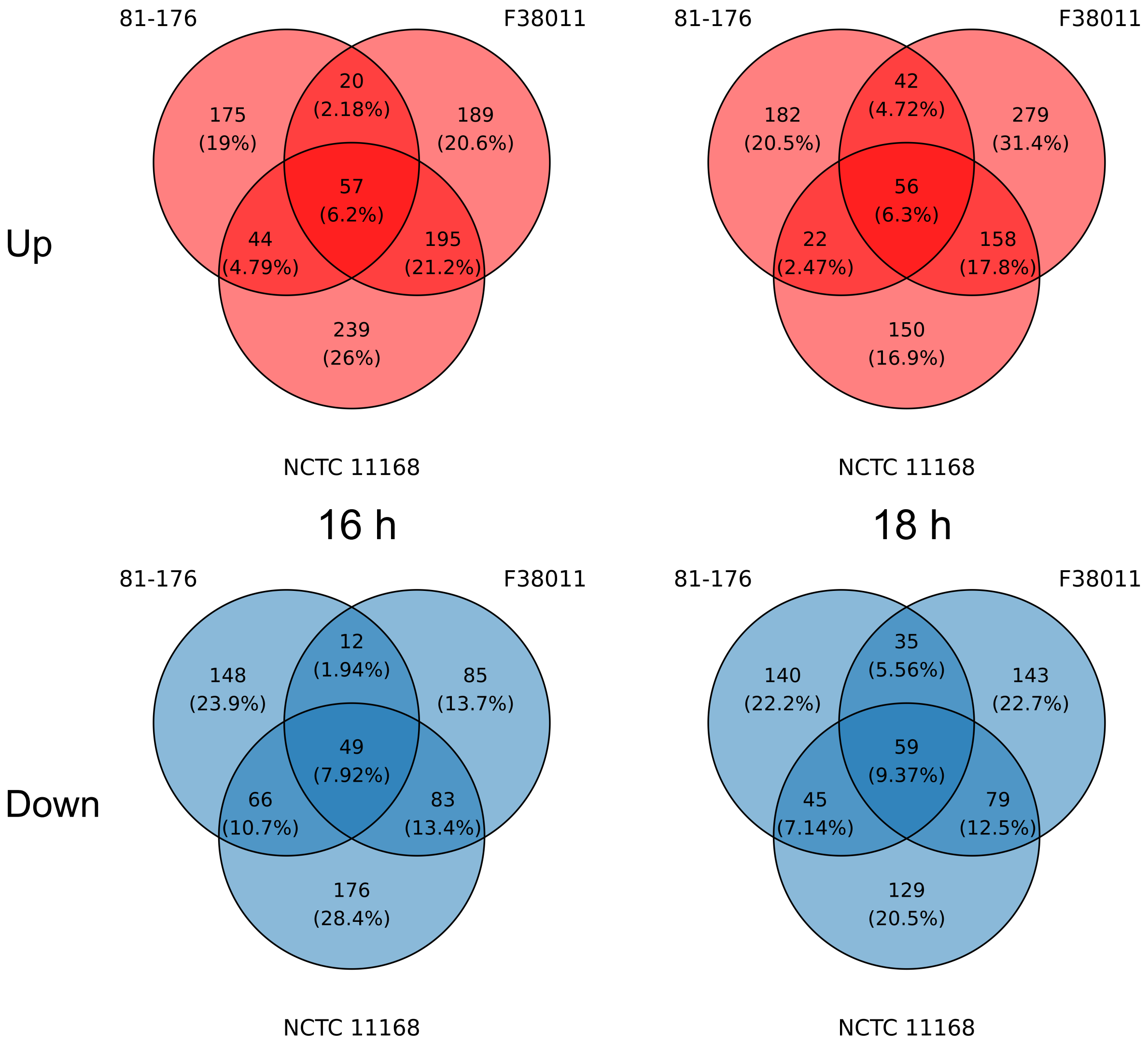

Supplementary Figure 4. Comparison of the number of genes upregulated and downregulated by each *C. jejuni* strain grown in MH with deoxycholate at 16 and 18 hours versus *C. jejuni* grown in MH for 12 hours. Genes that were significantly upregulated or downregulated had a Benjamini-Hochberg adjusted p value of less than 0.1 ($q < 0.1$) as determined by a Wald test implemented in DESeq2.

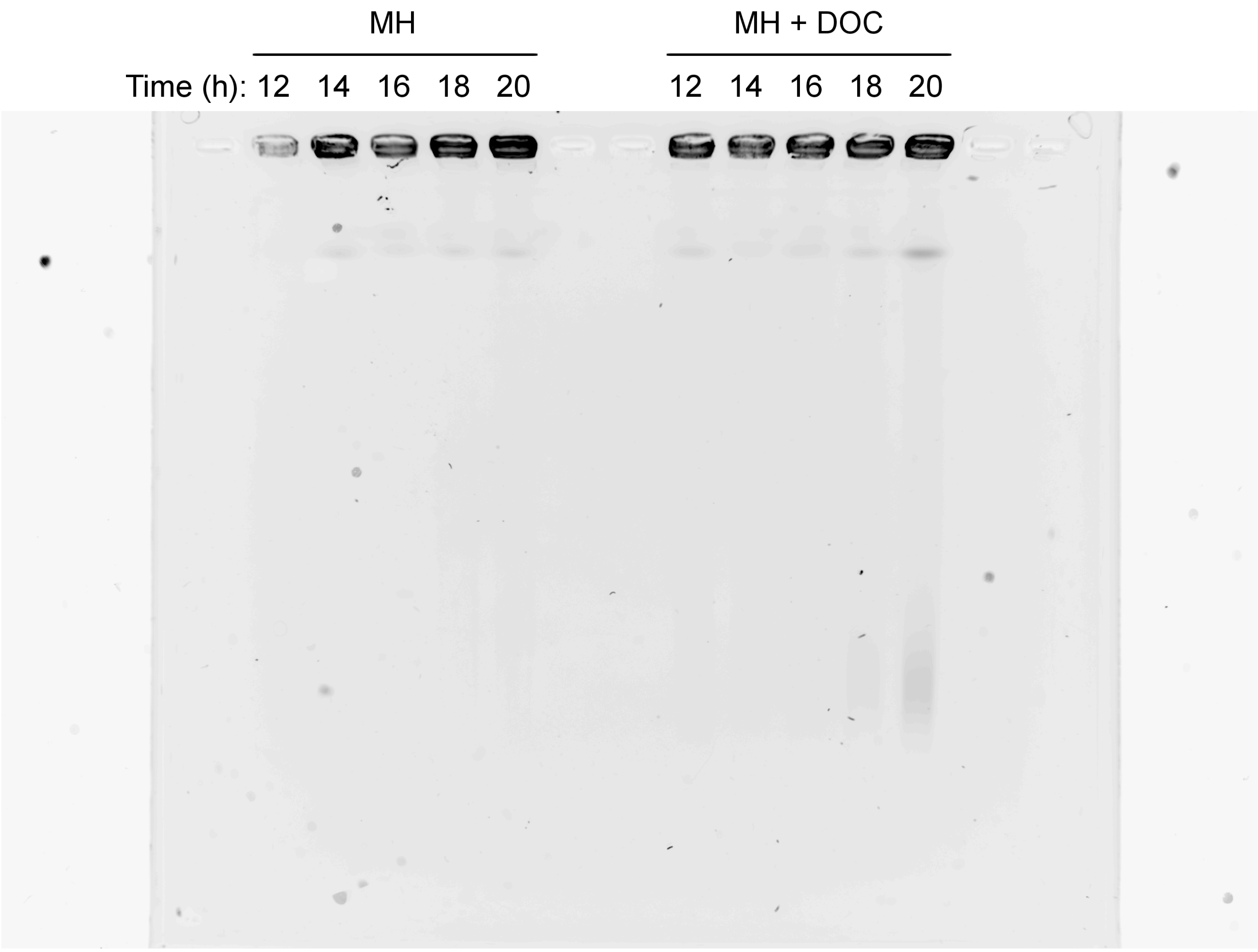

Supplementary Figure 5. Original image of Figure 4 (**a**), without contrast enhancement. DNA from *C. jejuni* strain 81-176 cultured in MH broth (left side of gel) and from *C. jejuni* cultured in MH broth with 0.05% (w/v) deoxycholate (DOC, right side of gel) was subjected to pulsed-field gel electrophoresis to evaluate DNA integrity. Intact DNA remains in the well, while DNA fragments migrate in the gel.
